# Supplementary material for: Smooth Interpolating Curves with Local Control and Monotone Alternating Curvature
Source: Comput Graph Forum. 2022 Oct 6;41(5):25–38. doi: 10.1111/cgf.14600 (PMC9827861; doi:10.1111/cgf.14600)
Supplement: Supplementary file 1 — Supplement Material [file CGF-41-25-s001.zip › Local-Smooth-Interpolating-MonoCurvature/extern/clothoids/submodules/Utils/docs/api-cpp/program_listing_file_Utils_rang.hxx.html]

Program Listing for File rang.hxx — Utils v1.2

### Navigation

- index
- toc
- Utils »
- Program Listing for File rang.hxx

# Program Listing for File rang.hxx¶

↰ Return to documentation for file (`Utils/rang.hxx`)

```
/*\

  Taken from: https://github.com/agauniyal/rang/blob/master/include/rang.hpp


\*/

#include <algorithm>
#include <atomic>
#include <cstdlib>
#include <cstring>
#include <iostream>

namespace rang {

  #ifndef DOXYGEN_SHOULD_SKIP_THIS
  using std::ostream;
  #endif

  /* For better compability with most of terminals do not use any style settings
   * except of reset, bold and reversed.
   * Note that on Windows terminals bold style is same as fgB color.
   */
  enum class style {
    reset     = 0,
    bold      = 1,
    dim       = 2,
    italic    = 3,
    underline = 4,
    blink     = 5,
    rblink    = 6,
    reversed  = 7,
    conceal   = 8,
    crossed   = 9
  };

  enum class fg {
    black   = 30,
    red     = 31,
    green   = 32,
    yellow  = 33,
    blue    = 34,
    magenta = 35,
    cyan    = 36,
    gray    = 37,
    reset   = 39
  };

  enum class bg {
    black   = 40,
    red     = 41,
    green   = 42,
    yellow  = 43,
    blue    = 44,
    magenta = 45,
    cyan    = 46,
    gray    = 47,
    reset   = 49
  };

  enum class fgB {
    black   = 90,
    red     = 91,
    green   = 92,
    yellow  = 93,
    blue    = 94,
    magenta = 95,
    cyan    = 96,
    gray    = 97
  };

  enum class bgB {
    black   = 100,
    red     = 101,
    green   = 102,
    yellow  = 103,
    blue    = 104,
    magenta = 105,
    cyan    = 106,
    gray    = 107
  };

  enum class control { // Behaviour of rang function calls
    Off   = 0,         // toggle off rang style/color calls
    Auto  = 1,         // (Default) autodect terminal and colorize if needed
    Force = 2          // force ansi color output to non terminal streams
  };
  // Use rang::setControlMode to set rang control mode

  enum class winTerm { // Windows Terminal Mode
    Auto   = 0,        // (Default) automatically detects wheter Ansi or Native API
    Ansi   = 1,        // Force use Ansi API
    Native = 2         // Force use Native API
  };
  // Use rang::setWinTermMode to explicitly set terminal API for Windows
  // Calling rang::setWinTermMode have no effect on other OS

  #ifndef DOXYGEN_SHOULD_SKIP_THIS

  namespace rang_implementation {

    #ifndef DOXYGEN_SHOULD_SKIP_THIS
    using std::atomic;
    using std::enable_if;
    using std::is_same;
    using std::ostream;
    using std::streambuf;
    #endif

    inline
    atomic<control> &
    controlMode() noexcept {
      static atomic<control> value(control::Auto);
      return value;
    }

    inline
    atomic<winTerm> &
    winTermMode() noexcept {
      static atomic<winTerm> termMode(winTerm::Auto);
      return termMode;
    }

    template <typename T>
    using enableStd = typename enable_if<
      is_same<T, rang::style>::value ||
      is_same<T, rang::fg>::value    ||
      is_same<T, rang::bg>::value    ||
      is_same<T, rang::fgB>::value   ||
      is_same<T, rang::bgB>::value,
      ostream &
    >::type;

    extern bool isTerminal( streambuf const * ) noexcept;
    extern bool supportsAnsi( streambuf const * ) noexcept;
    extern bool supportsColor() noexcept;

    #ifdef UTILS_OS_WINDOWS
    template <typename T>
    inline
    void
    setWinColorAnsi( ostream &os, T const value )   {
      os << "\033[" << static_cast<int>(value) << "m";
    }

    void setWinColorNative( ostream &os, enum rang::style value );
    void setWinColorNative( ostream &os, enum rang::bg    value );
    void setWinColorNative( ostream &os, enum rang::fg    value );
    void setWinColorNative( ostream &os, enum rang::bgB   value );
    void setWinColorNative( ostream &os, enum rang::fgB   value );

    template <typename T>
    inline
    enableStd<T>
    setColor( ostream &os, T const value ) {
      if (winTermMode() == winTerm::Auto) {
        if (supportsAnsi(os.rdbuf())) {
          setWinColorAnsi(os, value);
        } else {
          setWinColorNative(os, value);
        }
      } else if (winTermMode() == winTerm::Ansi) {
        setWinColorAnsi(os, value);
      } else {
        setWinColorNative(os, value);
      }
      return os;
    }
    #else
    template <typename T>
    inline
    enableStd<T>
    setColor( ostream &os, T const value ) {
      return os << "\033[" << static_cast<int>(value) << "m";
    }
    #endif

  }  // namespace rang_implementation

  template <typename T>
  inline
  rang_implementation::enableStd<T>
  operator << ( ostream &os, T const value ) {
    control const option = rang_implementation::controlMode();
    switch (option) {
      case control::Auto:
        return rang_implementation::supportsColor()
               && rang_implementation::isTerminal(os.rdbuf())
               ? rang_implementation::setColor(os, value)
               : os;
      case control::Force:
        return rang_implementation::setColor(os, value);
      default:
        return os;
    }
  }

  inline
  void
  setWinTermMode( rang::winTerm const value ) noexcept {
    rang_implementation::winTermMode() = value;
  }

  inline
  void
  setControlMode( control const value ) noexcept {
    rang_implementation::controlMode() = value;
  }
  #endif

}  // namespace rang
```

### Quick search

### Table of Contents

- rang - Colors for your Terminal
- Zstr
- C++ API

«
hide menu

menu
sidebar
»

### Navigation

- index
- toc
- Utils »
- Program Listing for File rang.hxx

© Copyright 2021, Enrico Bertolazzi.
Created using Sphinx 4.2.0.
